# Expression of PprI from *Deinococcus radiodurans* Improves Lactic Acid Production and Stress Tolerance in *Lactococcus lactis*

**DOI:** 10.1371/journal.pone.0142918

**Published:** 2015-11-12

**Authors:** Xiangrong Dong, Bing Tian, Shang Dai, Tao Li, Linna Guo, Zhongfang Tan, Zhen Jiao, Qingsheng Jin, Yanping Wang, Yuejin Hua

**Affiliations:** 1 Henan Provincial Key Laboratory of Ion Beam Bio-engineering, Zhengzhou University, Zhengzhou, 450052, China; 2 Key Laboratory for Nuclear-Agricultural Sciences of Chinese Ministry of Agriculture and Zhejiang Province, Institute of Nuclear-Agricultural Sciences, Zhejiang University, Hangzhou, 310029, China; 3 Institute of Crops and Utilization of Nuclear Technology, Zhejiang Academy of Agricultural Sciences, Hangzhou, 310021, China; Belgian Nuclear Research Centre SCK•CEN, BELGIUM

## Abstract

PprI is a general switch protein that regulates the expression of certain proteins involved in pathways of cellular resistance in the extremophilic bacterium *Deinococcus radiodurans*. In this study, we transformed *pprI* into *Lactococcus lactis* strain MG1363 using the lactococcal shuttle vector pMG36e and investigated its effects on the tolerance and lactic acid production of *L*. *lactis* while under stress. PprI was stably expressed in *L*. *lactis* as confirmed by western blot assays. *L*. *lactis* expressing PprI exhibited significantly improved resistance to oxidative stress and high osmotic pressure. This enhanced cellular tolerance to stressors might be due to the regulation of resistance-related genes (e.g., *recA*, *recO*, *sodA*, and *nah*) by *pprI*. Moreover, transformed *L*. *lactis* demonstrated increased lactic acid production, attributed to enhanced lactate dehydrogenase activity. These results suggest that *pprI* can improve the tolerance of *L*. *lactis* to environmental stresses, and this transformed bacterial strain is a promising candidate for industrial applications of lactic acid production.

## Introduction

Lactic acid is a glucose metabolite generally produced by lactic acid bacteria (LAB), e.g., *Lactococcus lactis*, and is present in sour milk, molasses, various fruits, and wines. Lactic acid has been widely used as a flavoring and preservative in foods and beverages and as a raw material for pharmaceuticals [1]. However, LAB are exposed to osmotic pressures including salt and acid stresses, during industrial fermentation and food processing, and the bacteria must overcome various physical and chemical barriers, such as oxidation in the gastrointestinal tract before they can provide a benefit to their host. In addition, facultative anaerobic LAB, which lack catalases, are sensitive to oxidative stress during manufacturing processes [2]. Modifying LAB strains via genetic engineering helps improve cellular tolerance and lactic acid production. For instance, Abdullah-Al-Mahin *et al*. expressed the DnaK protein from *Escherichia coli* in *L*. *lactis*, and the engineered strain exhibited significantly higher tolerances to acid (0.5% lactic acid, pH 5.47), salt (3% NaCl), and ethanol (5%) [3]. Tian *et al*. expressed small heat-shock protein (sHSP) gene derived from *S*. *thermophilus* in *L*. *lactis* and found that the genetically modified strain showed increased tolerance to acid, heat, ethanol, bile salts, and hydrogen peroxide [4]. When RecO from *Lactobacillus casei Zhang* was heterologously over-expressed in *L*. *lactis*, the biomass of the engineered strain increased by 22.03%, 37.04%, and 19.37%, respectively, when exposed to acidic (pH 5.0), salt (3% NaCl), and oxidative stress (0.1 mM H_2_O_2_) conditions [5]. Furthermore, RecO expression improved lactose dehydrogenase activity, thereby increasing lactic acid production by approximately 1.42-fold. These results suggest that the expression of exogenous genes might be an effective method for increasing cellular resistance and lactic acid production in lactobacilli under stress conditions.

A unique regulatory protein, PprI (DR0167, also named IrrE), was identified in the extremophilic bacterium *D*. *radiodurans* [6,7], which is known for its resistance to such stresses as irradiation, oxidants, and desiccation. PprI acts as a general switch for the expression of a number of proteins, including recombinase A (RecA), in pathways of cellular resistance [8], and disruption of the remarkably increases the sensitivity of the mutant to γ-rays, UV radiation, and mitomycin-C. Comparative proteomics analysis of the wild type and a *pprI* knock-out strain (YR1) identified 31 proteins that are significantly induced after irradiation in the presence of *pprI* [8,9]. These data suggest that *pprI* may play a key role in the regulation network of cellular resistance. The expression of *D*. *radiodurans pprI* enhanced the radioresistance of *E*. *coli* by approximately 1.6-fold, with significant increases in RecA expression [10]. The expression of PprI also significantly enhanced the free radical scavenging ability of *E*. *coli* by inducing the activity of catalase (KatG), indicating that exogenous expression of PprI promotes repair and protection pathways in *E*. *coli*. Moreover, transgenic *Brassica napus* expressing PprI can tolerate 350 mM (2%) NaCl, a concentration that inhibits the growth of almost all crop plants [11], suggesting that PprI can be used as a global regulator to improve stress tolerances in other organisms.

In this study, we expressed the *pprI* gene from *D*. *radiodurans* in *L*. *lactis* and investigated its effects on cellular tolerance and lactic acid production. The transcriptional regulations of resistance-related genes and lactate dehydrogenase gene by heterologous PprI was also evaluated.

## Materials and Methods

### Strains and growth conditions

Wild-type *D*. *radiodurans* strain R1 (lab stock) was grown in TGY broth (0.5% Bactotryptone, 0.1% glucose, 0.3% Bacto yeast extract) at 32°C with aeration or on TGY plates solidified with 1.5% agar. *E*. *coli* DH5a was purchased from Invitrogen (La Jolla, CA, USA). pGEM®-T Easy vector was purchased from Promega (Madison, WI, USA). *E*. *coli* cells were grown at 37°C in LB broth or on LB plates solidified with 1.5% agar and supplemented with 100 μg/mL ampicillin when grown under selection. *L*. *lactis* MG1363 was cultivated anaerobically at 30°C using GM17 medium (Difco Laboratories, Detroit, MI, USA) supplemented with 0.5% glucose. Bacterial growth was monitored by measuring the optical density (OD) at 600 nm.

### *PprI* cloning, vector construction, and transformation

The *pprI* gene was cloned using the following primers: 5′GAGCTCATGCCCAGTGCCAACGTCAGCCC3′ (upstream primer; the *Sac*I restriction enzyme site is underlined); 5′ AAGCTT GGGAAACCCGAAGGTCAGCTCG (downstream primer; the *Hind*III restriction enzyme site is underlined). The PCR conditions used were as follows: initial denaturation at 94°C for 5 min, 30 cycles of 94°C for 50 s, 60°C for 40 s and 72°C for 90 s, followed by 72°C for 5 min. The purified PCR product was cloned into pMD18-T vector and transformed into competent *E*. *coli* DH5a. For antibiotic selection, agarose plates containing 100 μg/mL ampicillin were used. *E*. *coli* cells were transformed using the modified CaCl_2_ technique. After screening using ampicillin-containing agarose plates, positive clones were selected; plasmids were then isolated, digested with restriction enzymes for identification, and sequenced. Clones with the correct sequence were isolated and digested with *Sac*I and *Hind*III to generate the *pprI* gene fragment with overhanging ends. The shuttle vector pMG36e was digested with the same enzymes, and the recovered vector fragment and target fragment were ligated overnight. The ligated product was transformed into competent *E*. *coli* MC1061, and positive clones were selected for expanded culture. Plasmids were isolated and digested with restriction enzymes for identification by gel electrophoresis using a 1% agarose gel at 120 V for 50 min, followed by DNA sequencing for verification (Shanghai Ruidi Co., China). The verified and correct clone was transformed into *L*. *lactis* MG1363 by electroporation to obtain the MG(PprI^+^) strain for further study. In parallel, an *L*. *lactis* strain transformed with the empty vector pMG36e was obtained as a control and designated MG(Vector).

### Western blot assay

To investigate the level of PprI expression in *L*. *lactis*, strains MG(PprI^+^) and MG(Vector) were grown in GM17 medium containing 500 mg/mL erythromycin at 30°C. Cell cultures at OD_600_ 1.0 were harvested by centrifugation at 4°C for 10 min. The cells were washed twice with ice-cold PBS buffer, resuspended in the same buffer and disrupted ultrasonically at 4°C for 99 cycles of 3 s each. The cell extract was obtained after centrifugation at 4°C for 10 min to remove cellular debris. The protein concentration was determined according to the Bradford method using bovine serum albumin as the standard. For protein analysis, the cell extract was mixed with a five-fold concentrated buffer; after being heated at 100°C for 10 min, a 15-μL aliquot of each sample was subjected to 12% SDS-PAGE. The proteins in the gel were transferred onto a polyvinylidene fluoride (PVDF) membrane (Amersham Pharmacia Biotech, Buckinghamshire, England) and incubated with a rabbit anti-PprI polyclonal antibody (rabbit IgG, laboratory stock). Chemiluminescent signals on the PVDF membrane were visualized and quantified using a Gel Imager System (Bio-Rad Laboratories, Hercules, CA, USA) [12].

### Tolerance assays of transformed bacteria under hydrogen peroxide and UV stress conditions

To determine the survival of bacteria under hydrogen peroxide stress, bacteria were cultured in LB medium to the exponential phase (OD_600_ approximately 0.5) and the stationary phase (OD_600_ approximately 1.5). H_2_O_2_ was added to final concentrations of 5, 10, and 20 mmol/L, and cells were collected every 20 min up to 1 h. MG(PprI^+^) without H_2_O_2_ treatment and MG(Vector) were used as the controls. The survival fraction was determined by counting the colonies on agarose plates.

Cell survival fractions when exposed to UV radiation were determined following a previously described method [13,14]. Bacteria were cultured in GM17 medium until OD_600_ reached approximately 1.0. 100 μL of cell culture were appropriately diluted in sterile phosphate-buffered saline (PBS) and plated on GM17 agar plates containing 500 μg/mL erythromycin and irradiated under UV light with a wavelength of 254 nm. Fluence rates were measured with a UV radiometer (TAINA Co. China). The plates were then incubated at 32°C for 36 h. Survival fraction was determined from the quotient of the number of colony formers after UV irradiation and the number of colony formers without UV irradiation. *F*10 value, indicating the fluence of UV irradiation resulting in 10% survival, was used for comparisons.

### Tolerance assays of transformed bacteria under osmotic pressure and acid shock

For the osmotic pressure experiment, bacteria were transferred into fresh GM17 culture medium to which 3% NaCl or 5% NaCl was added. The cells were cultured at 30°C, and sampling was performed every 4 h. Cell growth (OD_600_) was monitored using a Biophotometer (Eppendorf, Hamburg, Germany).

For the lactic acid tolerance assay, bacteria were transferred into GM17 culture media with lactic acid concentrations of 3%–5% (pH 5–6). To investigate the effects of pH (1–12) on cell survival, the bacteria were transferred into GM17 culture media with the pH adjusted by the addition of hydrochloric acid or sodium hydroxide. Sampling was performed every 4 h, and cell growth was monitored. As controls, MG(PprI^+^) without any stressors and MG(Vector) were also transferred into GM17 medium for culturing at 30°C.

### Analysis of lactic acid production

Cells grown to an OD_600_ of 1.0 were used as 2% inoculum (v/v) in fresh GM17 medium containing 500 μg/mL erythromycin and 0%, 3%, or 5% NaCl. Culturing was performed for 48 h at 30°C.

Samples withdrawn at different time intervals were centrifuged (10,000 x g for 10 min), and the supernatants were filtered through a 0.22 μm pore-size filter prior to analysis. The contents of lactic acid in the supernatants were assessed by HPLC using a Waters Alliance series 2695 separation module equipped with a 2487 dual-absorbance detector, which was controlled by Empower Pro software (Waters Corp., Milford, MA, USA). The HPLC conditions for lactic acid were as follows: Waters Nova-Pak C18 column (300 mm × 3.9 mm inner diameter, 4 mm particle size); mobile phase, 0.1 M KH_2_PO_4_ (pH adjusted to 2.5 with phosphoric acid); flow rate, 0.5 mL/min. Lactic acid was identified by retention time compared with a standard compound (lab stock). The amount of lactic acid was determined from the area under the peak detected at 240 nm using a calibration curve of lactic acid [15].

### Lactate dehydrogenase activity assay

Lactate dehydrogenase (LDH) activity in the cell extract was analyzed using a lactate dehydrogenase assay kit (Nanjing Jiancheng Bioengineering Institute, Jiangsu, China) following the manufacturer’s protocol. One unit of LDH activity was defined as the amount of enzyme required to convert the substrate to 1 μM pyruvate at 37°C in 15 min.

### Quantitative real-time PCR

QRT-PCR was performed according to a previously described method [16]. Total RNA was isolated using an RNA extraction kit (Invitrogen). The RNA samples were reverse-transcribed using a Protoscript First Strand cDNA Synthesis Kit (New England Bio-Labs, Beverly, MA, USA) as described in the manufacturer’s protocol. The expression of selected genes from different treatments was quantified by quantitative real-time PCR using a Bio-Rad Real-Time PCR System. The primers used were listed in S1 Table. First-strand cDNA was synthesized using a cDNA synthesis kit and PCR amplification was detected by SYBR Green fluorescence dye (Takara, Tokyo, Japan). Gene expression levels were normalized using housekeeping genes *tuf* and *gyrA* [17]. All real-time PCRs were performed as duplicates of three independent experiments.

### Statistical analysis

Results were assessed by Student’s *t*-test using PASW Statistics (Winwrap Basic.), and differences with *P*<0.05 was considered statistical significant.

## Results

### Expression of *pprI* in *L*. *lactis* enhanced cellular tolerance to hydrogen peroxide and UV

**Fig 1:**
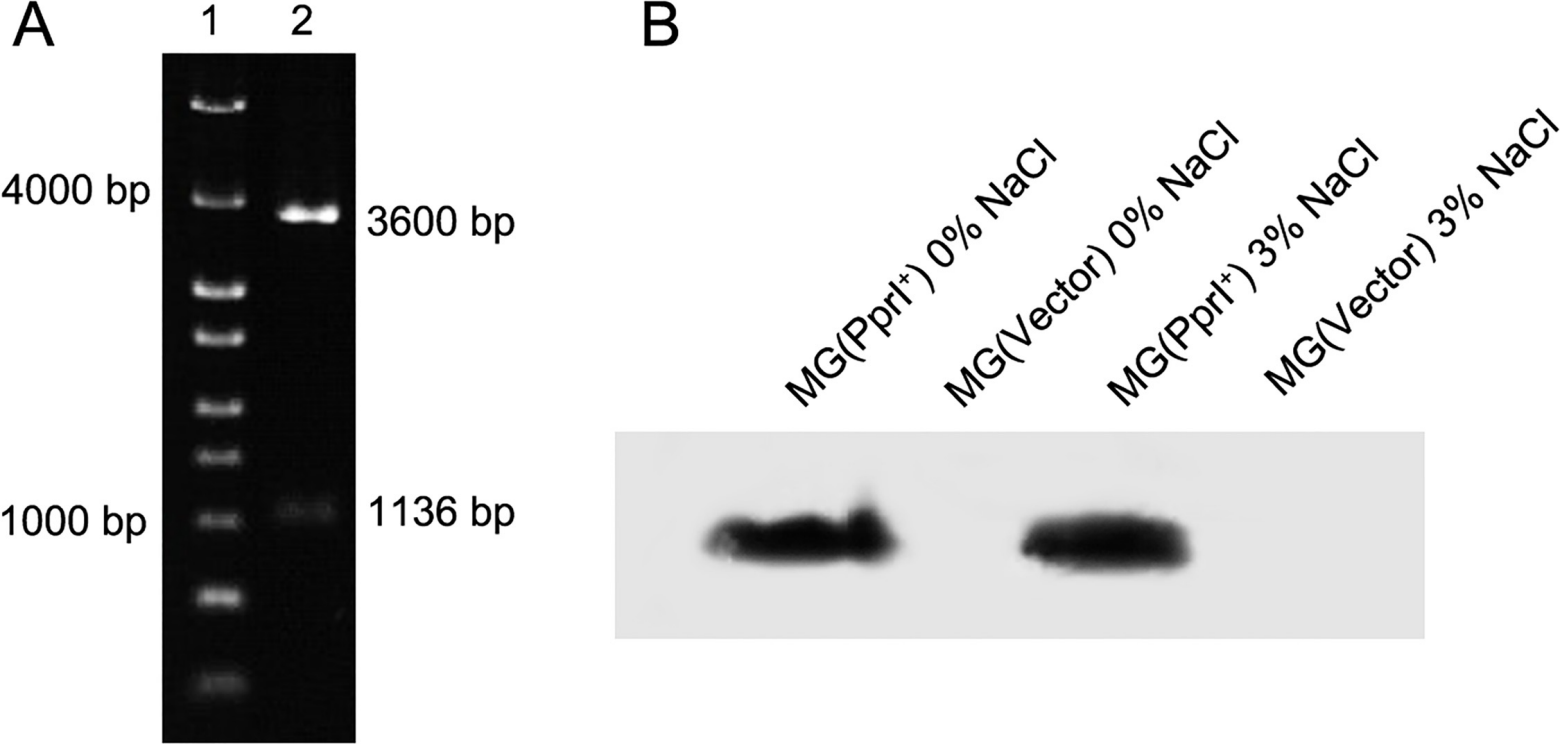
Expression of *pprI* in *L*. *lactis*. (A) Verification of the transformation of *pprI* into *L*. *lactis* MG(PprI^+^) via enzyme digestion of the plasmid:. Lane 1, DNA marker; lane 2, enzyme digestion of pMG36e isolated from *L*. *lactis* MG(PprI^+^) by *Sac*I and *Hind*III; (B) Western blot assay of the expression of PprI in *L*. *lactis* MG(PprI^+^) and MG(Vector).

The *pprI* gene was transformed into *L*. *lactis* strain MG1363 using the shuttle vector pMG36e, and *pprI* transformation was verified by enzyme digestion of pMG36e isolated from *L*. *lactis* MG(PprI^+^) (Fig 1A). The expression of PprI in *L*. *lactis* MG(PprI^+^) was confirmed by western blot assay compared with the control strain *L*. *lactis* MG(Vector) (Fig 1B). As shown in Fig 1B, *pprI* expression at 3% salt did not differ from that under salt-free conditions, consistent with a previous report that *pprI* expression in *D*. *radiodurans* is constitutive and does not change under various stresses [8,18].

**Fig 2:**
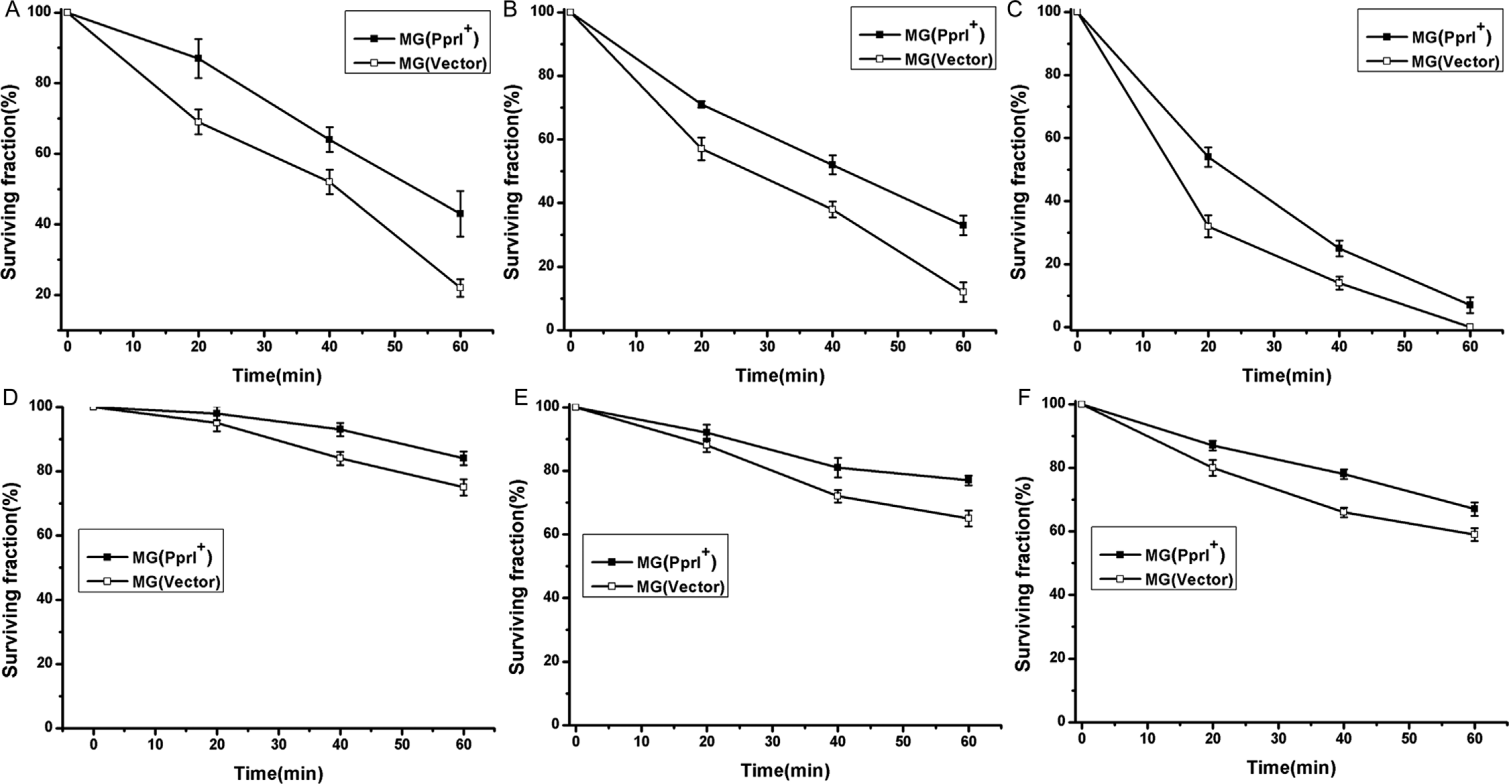
Survival fractions of *L*. *lactis* MG(PprI^+^) and MG(Vector) (carrying the empty vector pMG36e as a control) at different concentrations of H_2_O_2_. (A–C) Cells at the growth phase (OD_600_ approximately 0.5) treated with 5, 10, and 20 mM H_2_O_2_, respectively. (D–F) Cells at the stationary phase (OD_600_ approximately 1.0) treated with 5, 10, and 20 mM H_2_O_2_, respectively. Values are presented as the mean±standard deviation (SD) of three independent experiments.

**Fig 3:**
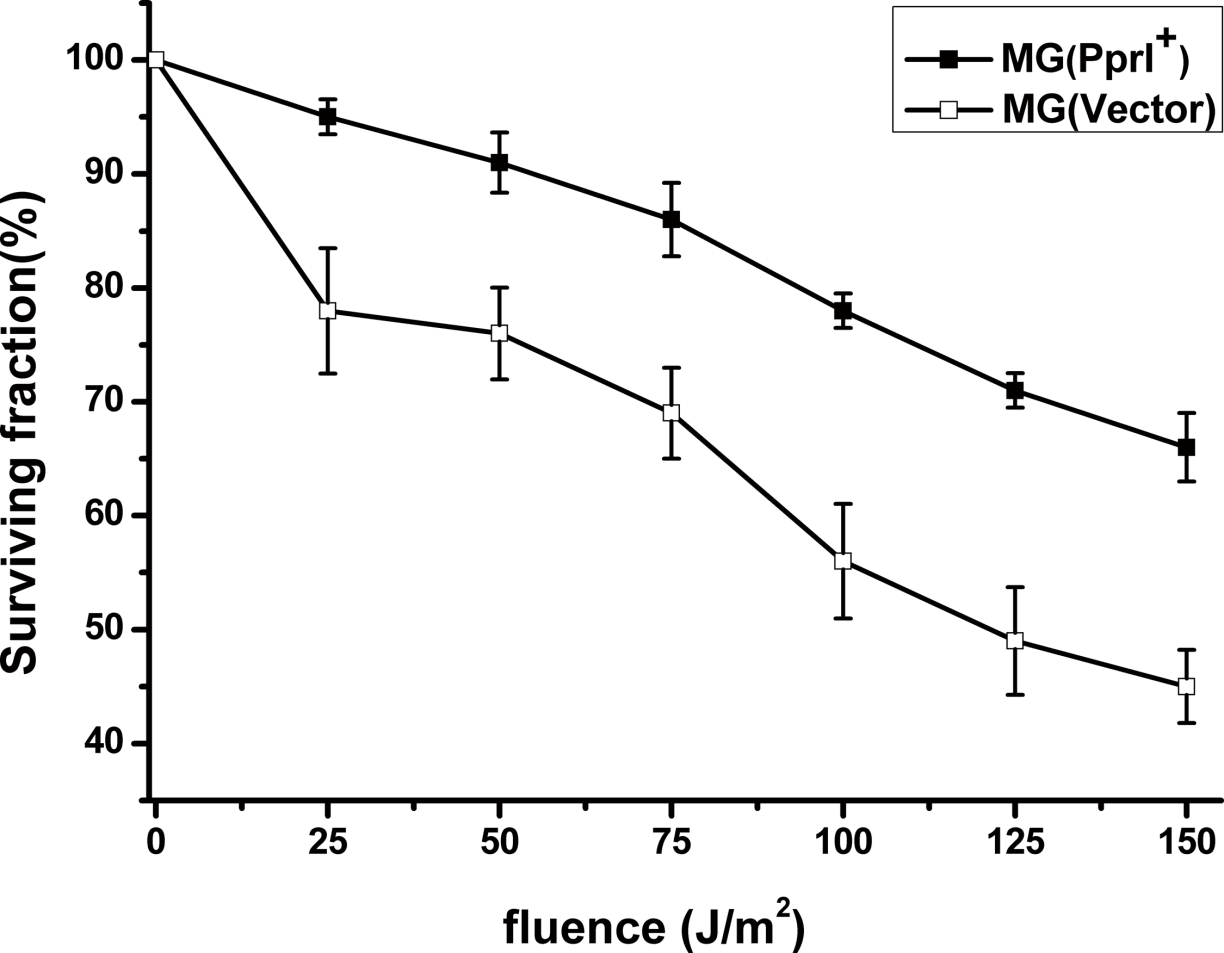
Survival fraction of *L*. *lactis* MG(PprI^+^) and MG(Vector) (carrying the empty vector pMG36e as a control) exposed to UV radiation. Cells plated on GM17 agar plates were treated with UV light at a wavelength at 254 nm. Values are presented as the mean±SD of three independent experiments.

The effects of PprI on the cellular tolerance of *L*. *lactis* to oxidative stresses (hydrogen peroxide and UV) were investigated (Figs 2 and 3). Fig 2 shows the survival of MG(PprI^+^) and MG(Vector) after treatment with different H_2_O_2_ concentrations at different growth phases. At the exponential growth phase (OD_600_ approximately 0.5), the survival fraction of MG(PprI^+^) was approximately 20% higher than that of the control MG(Vector) with 5 mM or 10 mM H_2_O_2_ treatment for 1 h (*P*<0.05)(Fig 2A and 2B). After treatment for 1 h at a higher H_2_O_2_ concentration (20 mM), no control bacteria survived, whereas a small amount of MG(PprI^+^) bacteria did survive (Fig 2C). However, at the stationary phase (OD_600_ approximately 1.0), the cell survival curves decreased more gradually at each H_2_O_2_ concentration compared to those during the exponential growth phase, suggesting that cells in the stationary phase are more resistant to oxidative stress. After treatment with 5, 10, and 20 mmol/L H_2_O_2_ for 1 h, the survival fractions of MG(PprI^+^) were approximately 85%, 80%, and 70% (Fig 2D–2F), respectively, which are all higher than those of the control MG(Vector)(*P*<0.05).

The gene-transformed strain MG(PprI^+^) and control strain MG(Vector) were exposed to UV at different fluences (Fig 3). The survival fraction of MG(PprI^+^) reached approximately 85% at UV fluence of 75 J/m^2^, compared with the control at approximately 70% survival. When the fluence was increased to 150 J/m^2^, the survival fraction of MG(PprI^+^) was approximately 25% higher than the control (*P*<0.05), suggesting that *L*. *lactis* bacteria transformed with PprI have enhanced resistance to UV radiation. The *F*10 value of MG(PprI^+^) was approximately 318 J/m^2^, which is higher than that of the control strain MG(Vector) (approximately 213 J/m^2^).

### Effects of PprI on *L*. *lactis* tolerance to osmotic pressures

**Fig 4:**
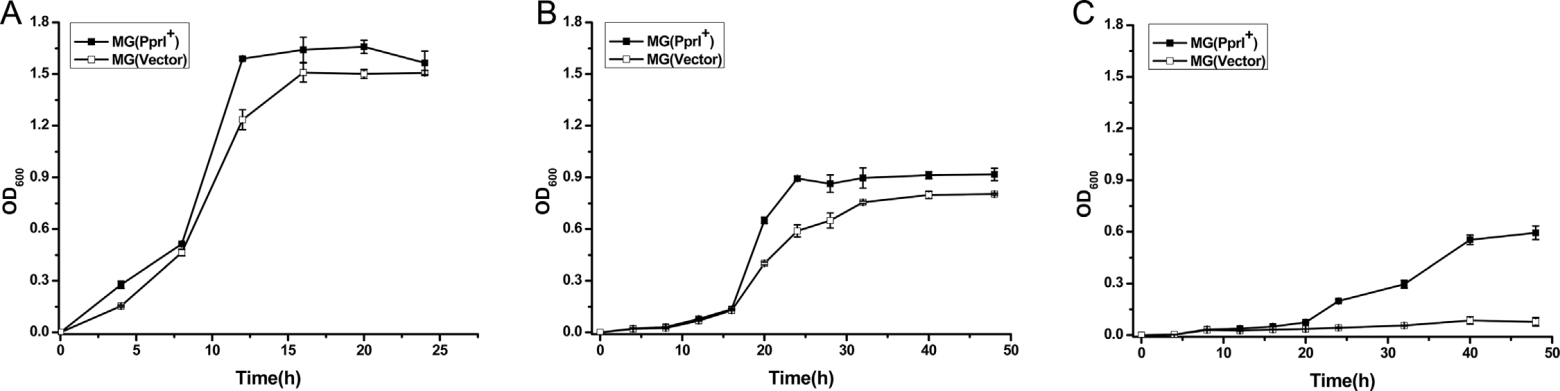
Cell growth of MG(PprI^+^) and MG(Vector) under NaCl stress. (A–C) Cells were cultivated at 30°C in the presence of 0%, 3%, and 5% NaCl, respectively. Values are presented as the mean±SD of three independent experiments.

**Fig 5:**
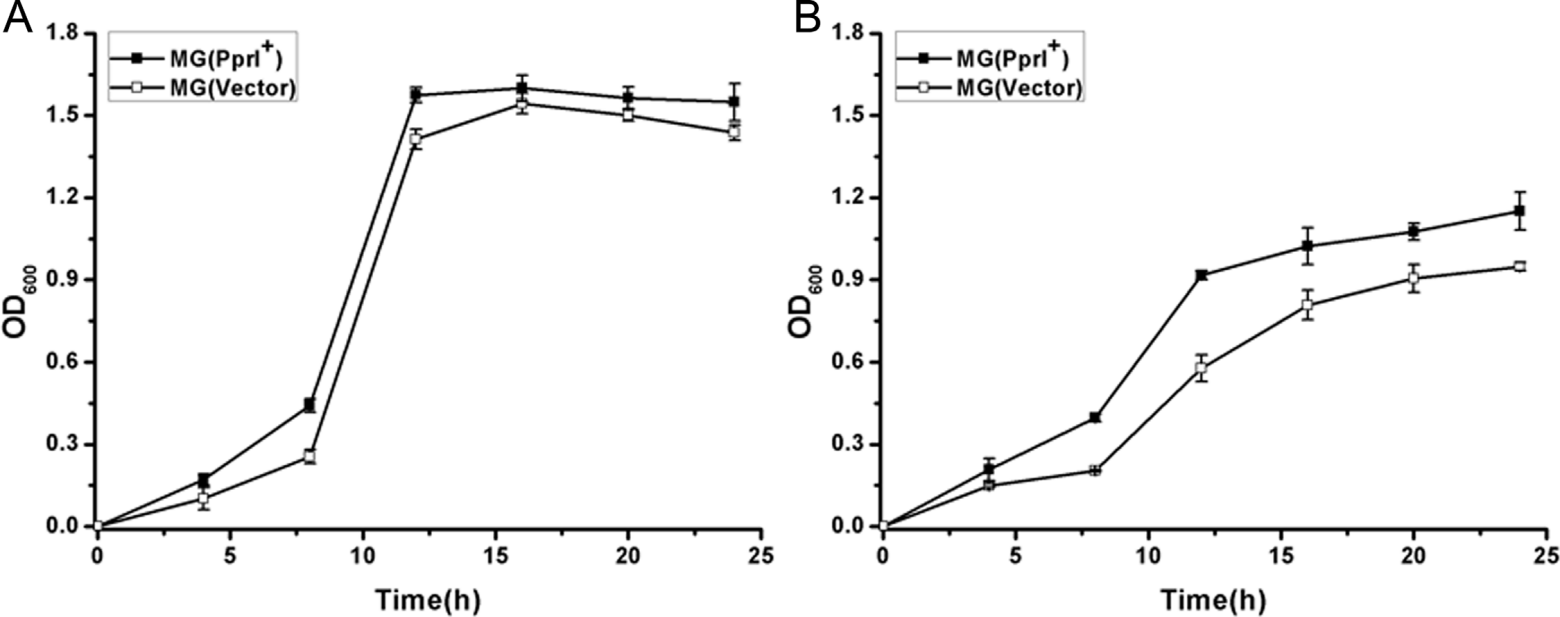
Cell growth curves of *L*. *lactis* in 3% lactic acid (A) and 5% lactic acid (B). Values are presented as the mean±SD of three independent experiments.

To determine the tolerances of MG(PprI^+^) and MG(Vector) at various osmotic pressures, cell growth in the presence of salt and acid stresses was monitored (Figs 4 and 5). In the absence of stress, no significant difference in cell growth was observed between MG(PprI^+^) and MG(Vector) (Fig 4A). However, at high concentrations of salt (3% NaCl, 5% NaCl), MG(PprI^+^) and MG(Vector) growth was inhibited compared with that in the absence of salt stress (Fig 4B and 4C). The OD_600_ of MG (PprI^+^) was seven times higher than that of the control when treated with 5% NaCl for 50 h (*P*<0.05) (Fig 4C); thus, MG(PprI^+^) showed significantly higher cellular tolerance than MG(Vector) under NaCl stress, suggesting that *pprI* enhances the resistance of *L*. *lactis* under salt stress.

*L*. *lactis* fermentation can be inhibited by the accumulation of lactic acid in the culture. Therefore, bacterial growth under high concentrations of lactic acid stress was measured. As shown in Fig 5, MG(PprI^+^) growth was higher than that of the control strain at 3% and 5% lactic acid, indicating that *L*. *lactis* expressing PprI exhibits enhanced tolerance to lactic acid.

**Fig 6:**
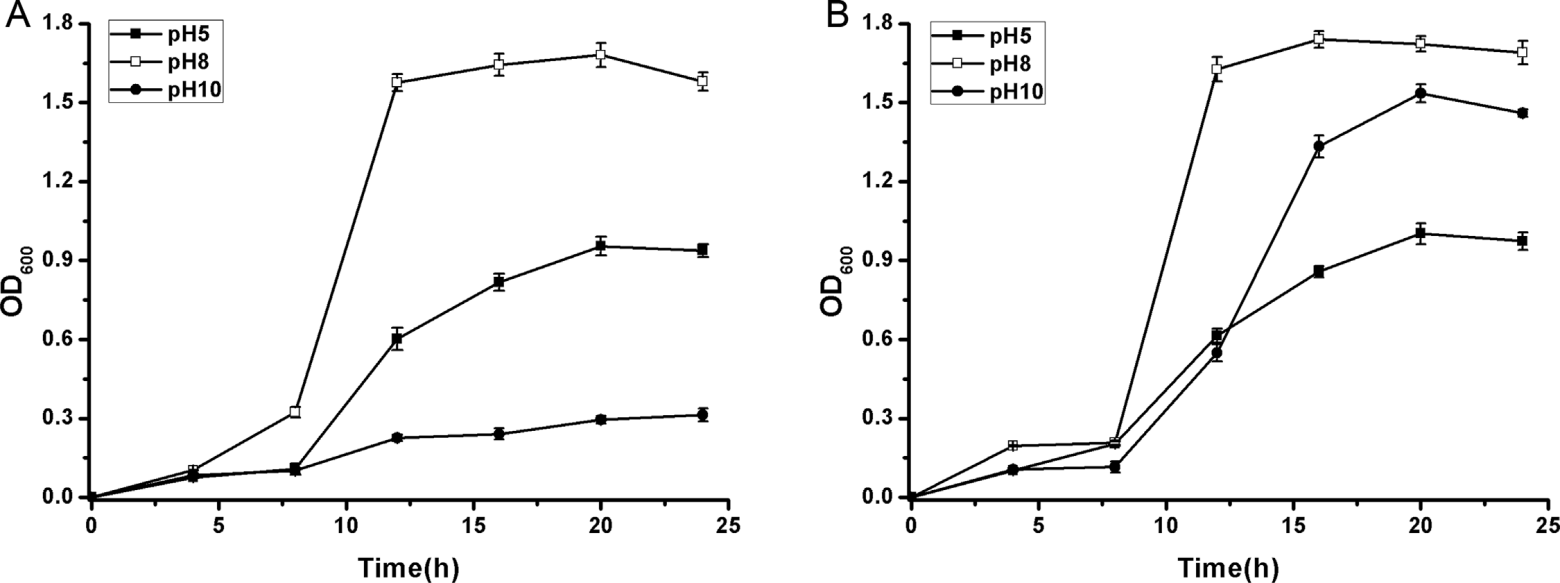
Cell growth curves of MG(Vector) (A) and MG(PprI^+^) (B) at pH 5, 8, and 10, respectively. Values are presented as the mean±SD of three independent experiments.

Because lactic acid accumulation results in pH changes in cell culture, cell growth at different pH values (5–10) was investigated (Fig 6). The *L*. *lactis* strains showed optimum cell growth at pH 8.0, whereas low pH (5.0) and high pH (10.0) had negative effects on cell growth. MG(PprI^+^) exhibited enhanced cellular tolerance at pH 5.0 and pH 10.0 (Fig 6B), compared with MG(Vector) (Fig 6A).

### Expression of *pprI* in *L*. *lactis* increased lactic acid production

**Fig 7:**
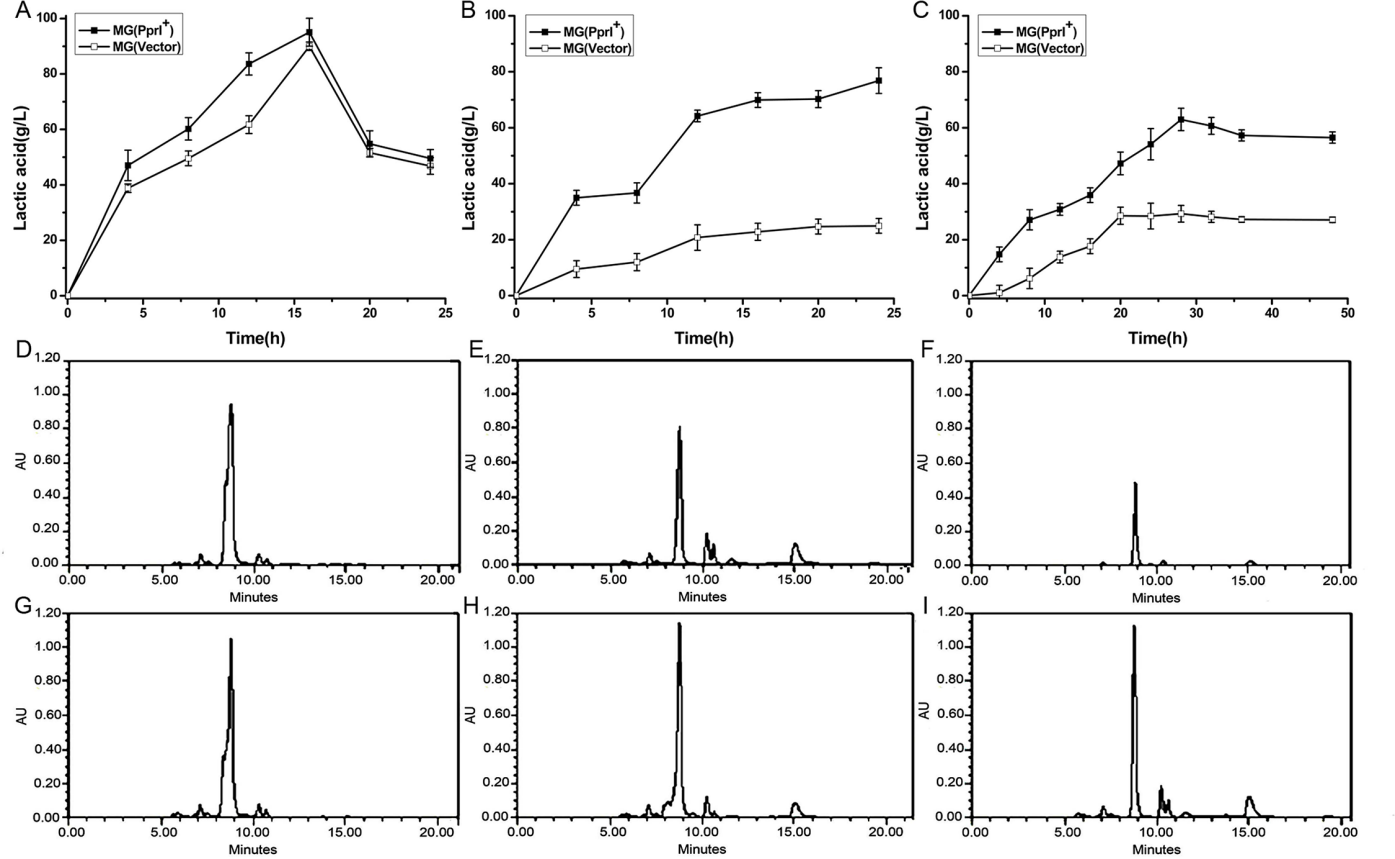
Lactic acid production of cell cultures from 0–24 h at 0%, 3%, and 5% NaCl. (A–C). Values are presented as the mean±SD of three independent experiments. (D–F) HPLC analysis of lactic acid content in MG(Vector) cultures at 0%, 3%, and 5% NaCl, respectively, after 18 h. (G–I) HPLC analysis of lactic acid content in MG(PprI^+^) cultures at 0%, 3%, and 5% NaCl, respectively, after 18 h. Representative HPLC results from three independent analyses are shown. Lactic acid was identified by retention time compared with standard compound (lab stock). The amount of lactic acid was determined from the area under the peak detected at 240 nm using a calibration curve of lactic acid.

Cell growth and lactic acid production of both the transformed and control strains under high-salt conditions were decreased compared to stress-free conditions (Figs 4 and 7), indicating that growth and lactic acid metabolism might have been affected under high-salt concentrations. However, MG(PprI^+^) showed a little higher lactic acid production than the control during the cell cultures from 5-15h under stress-free conditions (Fig 7A and 7D), and under salt stress, MG(PprI^+^) demonstrated significantly higher lactic acid production per cell culture volume than the control (Fig 7B, 7E, 7H and 7C, 7F, 7I). After 24 h, the lactic acid content in the cell supernatant was three times higher than the control at 3% NaCl (Fig 7B). At increased salt concentration (5% NaCl), the lactic acid content in the cell supernatant was not as high as that at 3% NaCl (Fig 7C), which might be due to the reduction of biomass at the higher salt concentration (Fig 4C). These results suggest that introduction of the *pprI* gene can induce lactic acid production in *L*. *lactis*, especially under salt stress. The transformant MG(PprI^+^) shows potential for application in lactic acid fermentation.

### PprI induced lactate dehydrogenase activity

**Fig 8:**
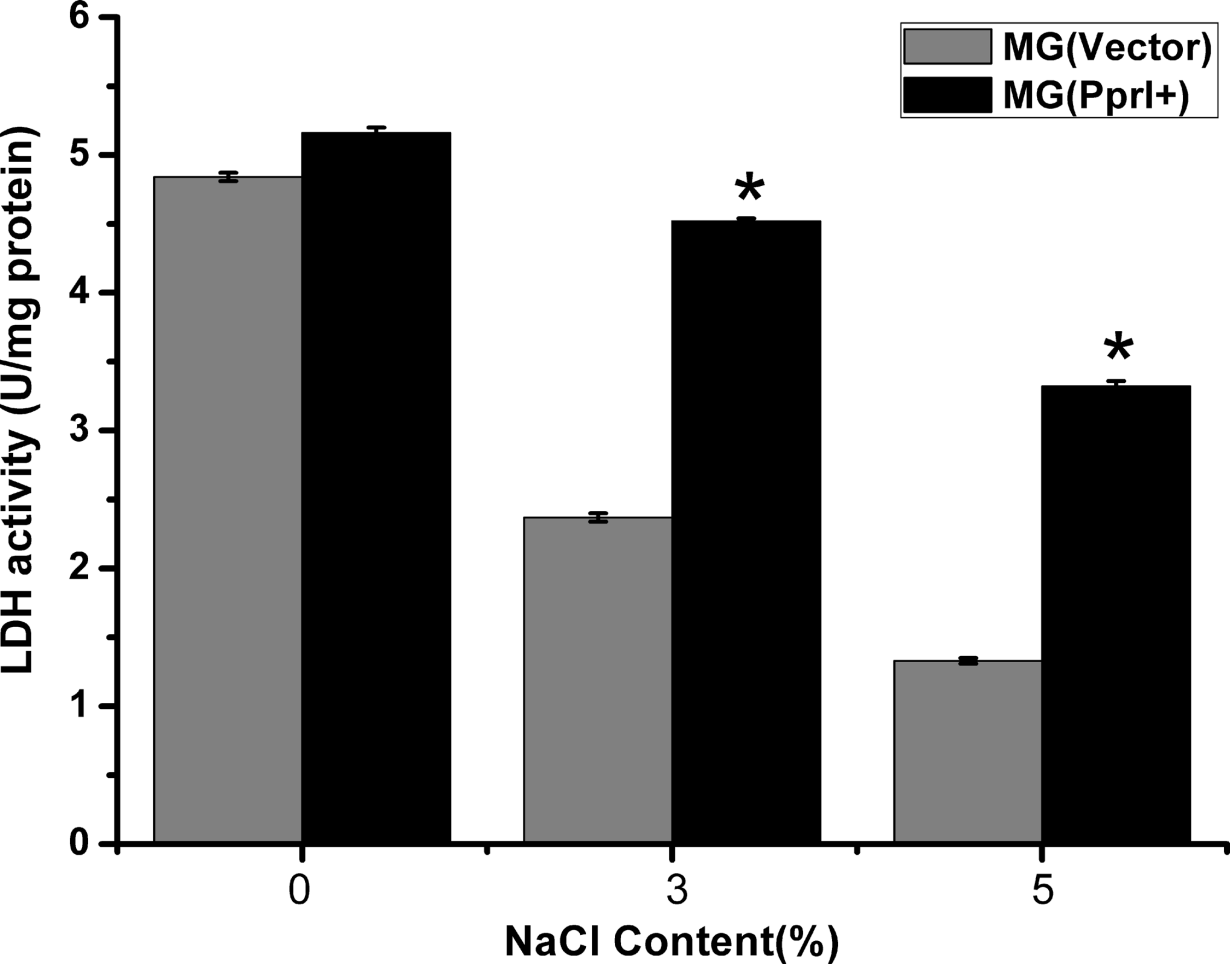
Lactate dehydrogenase activity of *L*. *lactis* under stress-free, 3% NaCl, and 5% NaCl conditions, respectively. *, *P*<0.05, indicating significant difference compared with the control strain.

To investigate how PprI increased lactic acid production in *L*. *lactis*, we measured the lactate dehydrogenase activity of *L*. *lactis* under stress-free, 3% NaCl, and 5% NaCl conditions. Under stress-free conditions, the lactate dehydrogenase activity of MG(PprI^+^) was only slightly higher than that of the control (Fig 8) but was 2.98-fold and 3.14-fold higher than that of the control at 3% and 5% NaCl (*P*<0.05), respectively. This result suggests that the lactate dehydrogenase activity of MG(PprI^+^) was significantly induced by the presence of PprI under high osmotic pressures. These results were in consistence with those of the lactic acid production assay.

### Transcriptional levels of *L*. *lactis* stress-related genes in the presence of PprI

**Fig 9:**
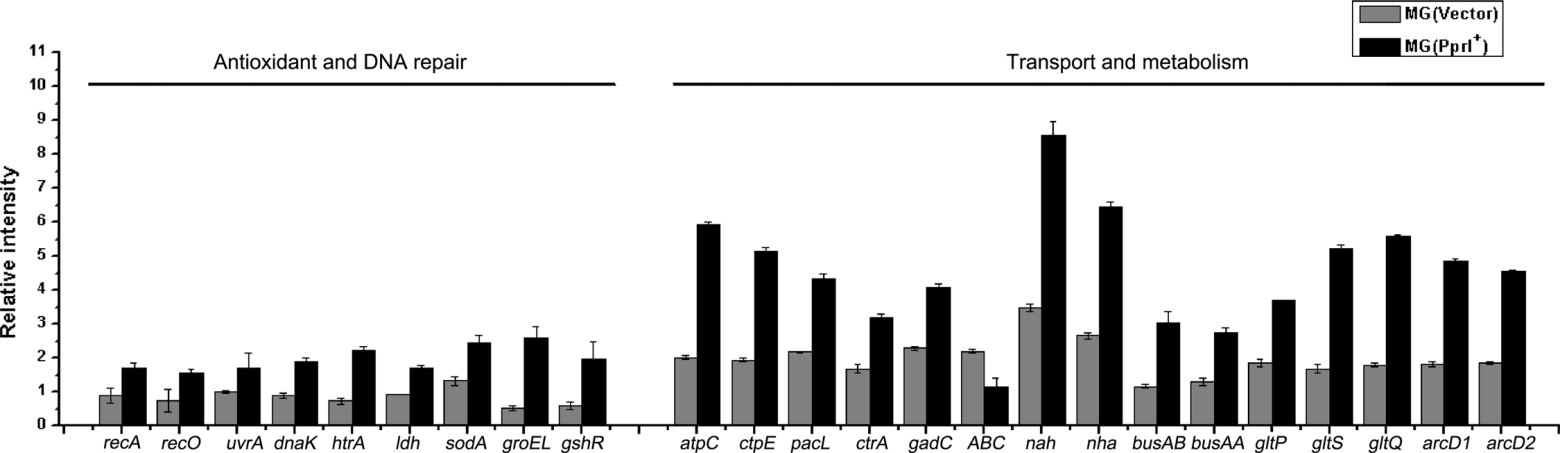
Real-time PCR analysis of gene expression under 3% NaCl salt stress compared with expression under no stress. Gene expression levels were normalized using the housekeeping genes *tuf* and *gyrA*. All real-time PCRs were performed as duplicates of three independent experiments.

PprI is a global regulator of various response genes. In the present study, the *L*. *lactis* transformant harboring the *pprI* gene demonstrated enhanced cellular tolerance to oxidative, salt, and acid stresses, as well as increased lactic acid production. Genes involved in oxidative, salt, and acid resistance were selected to evaluate changes in transcription to elucidate the roles of exogenous *pprI* in the transformant. The expressions of relevant genes in MG(PprI^+^) and the control strain was analyzed by RT-PCR. Under salt stress, some of the genes involved in ion metabolism, amino acid metabolism, and DNA repair were up-regulated in MG(PprI^+^). As shown in Fig 9, ion transport genes were significantly elevated under conditions of 3% salt. The Na^+^/H^+^ transport system (*nah* and *nha*) plays an important role in intracellular Na^+^ homeostasis [19,20], and expression of the two genes encoding Na^+^/H^+^ ion pumps (*nah* and *nha*) was increased by approximately two-fold in MG(PprI^+^) compared to the control. This result suggests that the transformant strain utilizes the Na^+^/H^+^ transport system to maintain intracellular Na^+^ homeostasis.

The transcription of an LDH gene homolog was induced by approximately two-fold in MG(PprI^+^) under salt stress (3% NaCl) (Fig 9), consistent with the results of induced lactate dehydrogenase activity in MG(PprI^+^). Certain genes involved in amino acid metabolism (*ctrA*) and glutamate metabolism (*gltQ* and *gltP*) were also moderately increased in MG(PprI^+^) compared to the control (S1 Table and Fig 9). The enhanced tolerance of MG(PprI^+^) to high environmental salt and acid concentrations indicated that *pprl* confers resistance to osmotic pressures to this *L*. *lactis*, possibly via the regulation of transport and metabolism systems of ions and metabolites.

In addition, the transcription of a series of stress-response genes, such as those encoding recombinant protein RecA, molecular chaperone DnaK, DNA repair protein RecO, and superoxide dismutase (SodA), was also induced in MG(PprI^+^) compared to the control strain.

The free radicals generated under high-salt stress induce the damage to DNA and proteins [21]. Free radical accumulation can be induced by salt stress and protection against oxidative injury is an important mechanism in salinity tolerance of plants and animals [22,23]. The accumulation of reactive oxygen species (ROS) induced by abiotic stresses, including salt stress, is toxic to cells [24]. Therefore, increased *sodA* and DNA repair genes might contribute to the enhanced cell tolerance of MG(PprI^+^) to oxidative stress.

## Discussion

LAB are gram-positive, low-GC, microaerophilic, non-sporulating, rod- or coccus-shaped bacteria that convert carbohydrates into lactic acid. *L*. *lactis* in particular is used for lactic acid production in the food and feed industries [1,25]. As LAB are anaerobes that lack catalase and are sensitive to salt and acid stress during manufacturing processes [26,27], the antioxidant mechanism present in LAB is an interesting issue deserving further investigation. In the present study, *L*. *lactis* MG1363 transformed with the *pprI* gene demonstrated strong tolerance to oxidative stress. *PprI*, identified in the extremophilic bacterium *D*. *radiodurans*, is a switch gene that regulates many resistance-related genes (*recA*, *ddrO*, *pprA*, etc.) [8]. It has been reported that the transformation of *L*. *lactis* with a *recO* homolog from *Lactobacillus casei* enhances the bacterium’s resistance to salt stress [5,28]. As the upstream regulator of *recO* and *recA* genes, the *pprI* gene product might function to further modulate bacterial tolerance. Transcription analysis showed that some of the genes involved in DNA repair, ion metabolism, and amino acid metabolism were indeed up-regulated by the presence of Pprl in MG(PprI^+^) under salt stress.

Previous studies on PprI by our lab and other groups revealed that this protein plays important roles in regulating functional proteins involved in DNA repair and protection pathways of *D*. *radiodurans*, including RecA, pleiotropic protein promoting DNA repair A (PprA) and catalase [6,7,9]. PprI was found to specifically bind to the promoters of *recA* and *pprA* but does not bind to nonspecific double-stranded DNA; in addition, and loss of the DNA binding activity of PprI resulted in the failure of RecA induction after radiation [8]. The status of PprI may change in response to DNA damage, and its conformation may also be altered. Recently, the regulatory mechanism of PprI with regard to its protease activity toward DdrO, a novel transcription factor that represses the expression of DNA damage response genes, was elucidated in *D*. *radiodurans* by our group [29] and in *D*. *deserti* by another group [30], respectively. After ionizing irradiation, PprI interacts with DdrO via the PprI-catalyzed cleavage of DdrO, which regulates DNA damage response (DDR) genes. Truncated DdrO then dissociates from its DNA target, relieving its repression of DDR genes. However, no DdrO homologs have been detected in the genome of *L*. *lactis*, suggesting that PprI might interact with an undiscovered protein with a function similar to DdrO or utilize an alternative regulatory mechanism in transformed *L*. *lactis*.

The biological actions of PprI when expressed in other organisms have been studied in recent years. Heterogeneous expression of the *pprI* gene significantly increased the resistance of *E*. *coli* to gamma irradiation and oxidative stress via PprI-induced RecA and the enzymatic activity of catalase [10]. In addition, the expression of PprI confers significantly enhanced salt tolerance in both *E*. *coli* and *B*. *napus* [11], and PprI regulates resistance proteins, including antioxidant enzymes as shown by comparative proteomic and transcriptomic analyses [11,31]. Moreover, PprI improves the growth and ethanol production of *Zymomonas mobilis* under ethanol and acid stresses by enhancing the activities of pyruvate decarboxylase and alcohol dehydrogenase [32]. In the present study, exogenous transformation of the *pprI* gene increased the antioxidant activity of *L*. *lactis*, and the induction of *sodA* might contribute to the enhanced cell tolerance of MG(PprI^+^) to oxidative stress. Expression of the *pprI* gene also significantly increased the resistance of *L*. *lactis* to UV radiation, which might be attributed to its induction of *recA* and *recO* that act in the repair of DNA damage.

*L*. *lactis* is generally exposed to high osmotic pressure, including salt and acid stresses, during lactic acid fermentation, such as in the preparation of cheese and pickles [33,34]. Additional sugar and salt are often needed during lactic acid fermentation, which might expose LAB to high osmotic pressure. Improvement of *L*. *lactis* tolerance to high osmotic pressures via genetic engineering is a promising strategy for the lactic acid fermentation industry. Here, we showed that *L*. *lactis* expressing the PprI protein displayed enhanced cellular tolerance to high osmotic pressure. The growth of *L*. *lactis* was inhibited by high concentrations of H_2_O_2_, NaCl, and lactic acid (Fig 2), which is consistent with a previous report on the inhibitory effects of lactic acid and oxidative metabolites in fermentation liquid [28]. High osmotic pressure (NaCl) during the production of cheese and pickles also affects the growth of *L*. *lactis* strains. However, transgenic *B*. *napus* expressing the PprI protein can tolerate salt stress [11]. The general response of LAB to such stress is to induce the expression of anti-osmotic stress proteins or to accumulate miscible solutes, such as K^+^, glutamic acid, proline, glycine, and betaine. The Na^+^/H^+^ transport systems (*nah* and *nha*) in MG(PprI^+^) were significantly elevated under salt stress, suggesting that *pprI* plays an important role in maintaining intracellular ion homeostasis. Genes involved in glutamine metabolism were also moderately increased in MG(PprI^+^) compared to the control, indicating that *pprI* increases the cellular resistance of LAB to osmotic pressure through its regulation of metabolite transport and metabolism. Although the detailed mechanisms underlying the increased tolerance of MG(PprI^+^) to osmotic pressure and oxidative stress are unclear, we hypothesized that PprI might directly regulate the transcription of related genes, or function indirectly as a protease interacting with an undiscovered protein. Future biochemical studies will be helpful in elucidating the regulatory roles of *pprI* in related pathways in LAB.

PprI-expressing *L*. *lactis* displayed an enhanced production of lactic acid due to increased lactate dehydrogenase activity, especially under salt stress. As the transcription level of lactate dehydrogenase gene was induced in the presence of *pprI*, the use of PprI-expressing *L*. *lactis* will most likely result in improved production of lactic acid in the food industry. Indeed, genetic engineering through the expression of genes from *D*. *radiodurans*, including *pprI* in *L*. *lactis* is a potential strategy to reduce the susceptibility of fermentation strains to osmotic pressure and oxidative stress during industrial fermentation and food processing. This genetically engineered strain with improved tolerance to environmental stresses is a promising candidate for industrial applications of lactic acid production and food fermentation.

## Supporting Information

S1 Table Primers used in this study. (DOC)

S2 Table Transcriptions of selected stress-related genes in MG(PprI^+^) under salt stress. (DOC)

## References

[1] Gaspar P, Carvalho AL, Vinga S, Santos H, Neves AR. From physiology to systems metabolic engineering for the production of biochemicals by lactic acid bacteria. Biotechnology advances. 2013;31(6):764–88. doi: 10.1016/j.biotechadv.2013.03.011 . 23567148 10.1016/j.biotechadv.2013.03.011

[2] Corcoran BM, Stanton C, Fitzgerald G, Ross Pages RP. Life under stress: the probiotic stress response and how it may be manipulated. Curr Pharm Des. 2008; 18: 1382–1399. 10.2174/138161208784480225 18537661

[3] Abdullah Al M, Sugimoto S, Higashi C, Matsumoto S, Sonomoto K. Improvement of multiple-stress tolerance and lactic acid production in *Lactococcus lactis* NZ9000 under conditions of thermal stress by heterologous expression of *Escherichia coli DnaK* . Applied and environmental microbiology. 2010;76(13):4277–85. doi: 10.1128/AEM.02878-09 . 20453133 10.1128/AEM.02878-09 PMC2897460

[4] Tian H, Tan J, Zhang L, Gu X, Xu W, Guo X, et al Increase of stress resistance in *Lactococcus lactis* via a novel food-grade vector expressing a shsp gene from *Streptococcus thermophilus* . Braz J Microbiol. 2012; 43(3): 1157–1164. doi: 10.1590/S1517-838220120003000043 24031940 10.1590/S1517-838220120003000043 PMC3768874

[5] Wu C, Zhang J, Du G, Chen J. Heterologous expression of *Lactobacillus casei* RecO improved the multiple-stress tolerance and lactic acid production in *Lactococcus lactis* NZ9000 during salt stress. Bioresource technology. 2013;143:238–41. doi: 10.1016/j.biortech.2013.05.050 . 23796607 10.1016/j.biortech.2013.05.050

[6] Earl AM, Mohundro MM, Mian IS, Battista JR. The IrrE Protein of *Deinococcus radiodurans* R1 Is a Novel Regulator of *recA* Expression. Journal of bacteriology. 2002;184(22):6216–24. doi: 10.1128/jb.184.22.6216–6224.2002 12399492 10.1128/JB.184.22.6216-6224.2002 PMC151961

[7] Hua Y, Narumi I, Gao G, Tian B, Satoh K, Kitayama S, et al PprI: a general switch responsible for extreme radioresistance of *Deinococcus radiodurans* . Biochemical and Biophysical Research Communications. 2003;306(2):354–60. doi: 10.1016/s0006-291x(03)00965-3 12804570 10.1016/s0006-291x(03)00965-3

[8] Lu H, Chen H, Xu G, Shah AM, Hua Y. DNA binding is essential for PprI function in response to radiation damage in *Deinococcus radiodurans* . DNA repair. 2012;11(2):139–45. doi: 10.1016/j.dnarep.2011.10.013 . 22051194 10.1016/j.dnarep.2011.10.013

[9] Lu H, Gao G, Xu G, Fan L, Yin L, Shen B, et al *Deinococcus radiodurans* PprI switches on DNA damage response and cellular survival networks after radiation damage. Mol Cell Proteomics. 2009; 8(3): 481–494. doi: 10.1074/ 18953020 10.1074/mcp.M800123-MCP200 PMC2649811

[10] Gao G, Tian B, Liu L, Sheng D, Shen B, Hua Y. Expression of *Deinococcus radiodurans* PprI enhances the radioresistance of *Escherichia coli* . DNA repair. 2003;2(12):1419–27. doi: 10.1016/j.dnarep.2003.08.012 14642569 10.1016/j.dnarep.2003.08.012

[11] Pan J, Wang J, Zhou Z. IrrE, a global regulator of extreme radiation resistance in *Deinococcus radiodurans*, enhances salt tolerance in *Escherichia coli* and *Brassica napus* . PLoS One 2009; 4(2): e4422 doi: 10.1371/journal.pone.0004422.g001 19204796 10.1371/journal.pone.0004422 PMC2635966

[12] Gu W, Xia Q, Yao J, Fu S, Guo J, Hu X. Recombinant expressions of sweet plant protein mabinlin II in *Escherichia coli* and food-grade *Lactococcus lactis* . World journal of microbiology & biotechnology. 2015;31(4):557–67. doi: 10.1007/s11274-015-1809-2 . 25649203 10.1007/s11274-015-1809-2

[13] Wang L, Xu G, Chen H, Zhao Y, Xu N, Tian B, et al DrRRA: a novel response regulator essential for the extreme radioresistance of *Deinococcus radiodurans* . Molecular microbiology. 2008;67(6):1211–22. doi: 10.1111/j.1365-2958.2008.06113.x . 18208531 10.1111/j.1365-2958.2008.06113.x

[14] Bauermeister A, Bentchikou E, Moeller R, Rettberg P. Roles of PprA, IrrE, and RecA in the resistance of *Deinococcus radiodurans* to germicidal and environmentally relevant UV radiation. Archives of microbiology. 2009;191(12):913–8. doi: 10.1007/s00203-009-0522-7 . 19882142 10.1007/s00203-009-0522-7

[15] Fu RY, Chen J, Li Y. Heterologous leaky production of transglutaminase in *Lactococcus lactis* significantly enhances the growth performance of the host. Applied and environmental microbiology. 2005;71(12):8911–9. doi: 10.1128/AEM.71.12.8911–8919.2005 . 16332889 10.1128/AEM.71.12.8911-8919.2005 PMC1317339

[16] Sun H, Li M, Xu G, Chen H, Jiao J, Tian B, et al Regulation of MntH by a dual Mn(II)- and Fe(II)-dependent transcriptional repressor (DR2539) in *Deinococcus radiodurans* . PloS one. 2012;7(4):e35057 doi: 10.1371/journal.pone.0035057 . 22523570 10.1371/journal.pone.0035057 PMC3327659

[17] Ulve VM, Monnet C, Valence F, Fauquant J, Falentin H, Lortal S. RNA extraction from cheese for analysis of in situ gene expression of *Lactococcus lactis* . Journal of applied microbiology. 2008;105(5):1327–33. doi: 10.1111/j.1365-2672.2008.03869.x . 18795980 10.1111/j.1365-2672.2008.03869.x

[18] Gao G, Le D, Huang L, Lu H, Narumi I, Hua Y. Internal promoter characterization and expression of the *Deinococcus radiodurans pprI-folP* gene cluster. FEMS Microbiol Lett. 2006;257(2):195–201. doi: 10.1111/j.1574-6968.2006.00169.x . 16553853 10.1111/j.1574-6968.2006.00169.x

[19] Castle AM, Macnab RM, Shulman RG. Coupling between the sodium and proton gradients in respiring *Escherichia coli* cells measured by ^23^Na and ^31^P nuclear magnetic resonance. J Biol Chem. 1986; 261: 7797–7806. 3011799

[20] Epstein W, Schultz S. Cation Transport in *Escherichia coli* . J Gen Physiol. 1965; 49: 221–234. 19873561 10.1085/jgp.49.2.221 PMC2195484

[21] Fini A, Guidi L, Giordano C, Baratto MC, Ferrini F, Brunetti C, et al Salinity stress constrains photosynthesis in *Fraxinus ornus* more when growing in partial shading than in full sunlight: consequences for the antioxidant defence system. Annals of botany. 2014;114(3):525–38. doi: 10.1093/aob/mcu130 . 25006177 10.1093/aob/mcu130 PMC4204663

[22] Chinnusamy V, Jagendorf A, Zhu JK. Understanding and Improving Salt Tolerance in Plants. Crop Science. 2005; 45:437–448.

[23] Freire CA, Togni VG, Hermes-Lima M. Responses of free radical metabolism to air exposure or salinity stress, in crabs (*Callinectes danae* and *C*. *ornatus*) with different estuarine distributions. Comparative biochemistry and physiology Part A, Molecular & integrative physiology. 2011;160(2):291–300. doi: 10.1016/j.cbpa.2011.06.024 . 21742051 10.1016/j.cbpa.2011.06.024

[24] Alscher RG, Erturk N, Heath LS. Role of superoxide dismutases (SODs) in controlling oxidative stress in plants. Journal of Experimental Botany. 2002; 53:1331–1341. 11997379

[25] Douillard FP, de Vos WM. Functional genomics of lactic acid bacteria: from food to health. Microbial cell factories. 2014;13 Suppl 1:S8 doi: 10.1186/1475-2859-13-S1-S8 . 25186768 10.1186/1475-2859-13-S1-S8 PMC4155825

[26] Wu C, Zhang J, Chen W, Wang M, Du G, Chen J. A combined physiological and proteomic approach to reveal lactic-acid-induced alterations in *Lactobacillus casei* Zhang and its mutant with enhanced lactic acid tolerance. Applied microbiology and biotechnology. 2012;93(2):707–22. doi: 10.1007/s00253-011-3757-6 . 22159611 10.1007/s00253-011-3757-6

[27] Wu C, Zhang J, Wang M, Du G, Chen J. *Lactobacillus casei* combats acid stress by maintaining cell membrane functionality. Journal of industrial microbiology & biotechnology. 2012;39(7):1031–9. doi: 10.1007/s10295-012-1104-2 . 22366811 10.1007/s10295-012-1104-2

[28] Zhang M, Chen J, Zhang J, Du G. The effects of RecO deficiency in *Lactococcus lactis* NZ9000 on resistance to multiple environmental stresses. Journal of the science of food and agriculture. 2014;94(15):3125–33. doi: 10.1002/jsfa.6662 . 24648035 10.1002/jsfa.6662

[29] Wang Y, Xu Q, Lu H, Lin L, Wang L, Xu H, et al Protease activity of PprI facilitates DNA damage response: Mn2+-dependence and substrate sequence-specificity of the proteolytic reaction. PloS one. 2015;10(3):e0122071 doi: 10.1371/journal.pone.0122071 . 25811789 10.1371/journal.pone.0122071 PMC4374696

[30] Ludanyi M, Blanchard L, Dulermo R, Brandelet G, Bellanger L, Pignol D, et al Radiation response in *Deinococcus deserti*: IrrE is a metalloprotease that cleaves repressor protein DdrO. Molecular microbiology. 2014;94(2):434–49. doi: 10.1111/mmi.12774 . 25170972 10.1111/mmi.12774

[31] Zhou Z, Zhang W, Chen M, Pan J, Lu W, Ping S, et al Genome-wide transcriptome and proteome analysis of *Escherichia coli* expressing IrrE, a global regulator of *Deinococcus radiodurans* . Molecular bioSystems. 2011;7(5):1613–20. doi: 10.1039/c0mb00336k . 21380435 10.1039/c0mb00336k

[32] Ying Z, Ma R, Zhao Z, Zhou ZF, Lu W, Zhang W, et al *IrrE*, an Exogenous Gene from *Deinococcus radiodurans*, improves the Growth of and Ethanol Production by a *Zymomonas mobilis* Strain under Ethanol and Acid Stresses. Journal of Microbiology and Biotechnology. 2010;20(7):1156–62. doi: 10.4014/jmb.0912.12036 20668411 10.4014/jmb.0912.12036

[33] Karimi R, Mortazavian AM, Karami M. Incorporation of *Lactobacillus casei* in Iranian ultrafiltered Feta cheese made by partial replacement of NaCl with KCl. Journal of dairy science. 2012;95(8):4209–22. doi: 10.3168/jds.2011-4872 . 22818434 10.3168/jds.2011-4872

[34] Aso Y, Takeda A, Sato M, Takahashi T, Yamamoto T, Yoshikiyo K. Characterization of lactic acid bacteria coexisting with a nisin Z producer in *Tsuda*-turnip pickles. Current microbiology. 2008;57(1):89–94. doi: 10.1007/s00284-008-9161-5 . 18437458 10.1007/s00284-008-9161-5

