# Supplementary material for: Expression of PprI from Deinococcus radiodurans Improves Lactic Acid Production and Stress Tolerance in Lactococcus lactis
Source: PLoS One. 2015 Nov 12;10(11):e0142918. doi: 10.1371/journal.pone.0142918 (PMC4643010; doi:10.1371/journal.pone.0142918)
Supplement: S1 Table — (DOC) [file pone.0142918.s001.doc]

**S1 Table. Primers used in this study**

| Primer | Sequence |
| --- | --- |
| pprI- F | GAGCTCATGCCCAGTGCCAACGTCAGCCC3 |
| pprI- R | AAGCTT GGGAAACCCGAAGGTCAGCTCG |
| pprI-RT F | CGCTGGCCCAAGCACAGAA |
| pprI-RT R | CGTCCACCTCCGCCTTCATT |
| recA -RT F | ATTACTTCTGGAGCGGTTGACTTGG |
| recA -RT R | CAAGTTTACGCATGGCTTGTGACAT |
| recO-RT F | CGATGCAGCCATTTCTGATAATCAA |
| recO-RT R | TGAAACCCCAAAACGATGAAGAACT |
| uvrA-RT F | GAACTCCCAGAAAAAACACGTTTGC |
| uvrA-RT R | TACCTTCTTTCACGACAATCCGGTC |
| dnaK-RT F | TCTTTGATGTTCTTGCAACAGCAGG |
| dnaK-RT R | AATGCCATTTTGTCTTGACCAAGGT |
| htrA-RT F | ATTGCAGTCGGCTCACCTTTAGGTA |
| htrA-RT R | AATCAAGGCTCCACCTGAGTTACCA |
| ldh-RT F | GTAGGTTCATCATACGCTTTTGCCC |
| ldh-RT R | CACCAGAAGTCAAGACAACGAGGTC |
| SodA-RT F | TGATGGTTCTGAAAACCATGCTGAC |
| SodA-RT R | TCAATTTGCCAGCTTCATCAACAAC |
| groEL-RT F | CAGCTGAAACAGCAGTTGCTTCAAT |
| groEL-RT R | CAAGCTCTGTTTGCATTCCTTTTGA |
| gshR-RT F | TGACTGATGGAGCTGGTTTTGAACA |
| gshR-RT R | CCCATGGCATAAATTTTAGGATTGC |
| atpC-RT F | TTTGCCAAACATGATTTCGACAATC |
| atpC-RT R | CAATATCACGATTACGTTCGGCAGA |
| ctpE-RT F | GTCGTGTGGTCAATAATGTTCAGCG |
| ctpE-RT R | TCCTGGAATCCCAATGGTGAATAAA |
| pacL-RT F | TTATTGACCCTCCTCGGGAAGAAGT |
| pacL-RT R | CCTAAATCATAAGCAATTGCTCGGG |
| ctrA-RT F | GGGATGCATCCTTACTCAGCCTATG |
| ctrA-RT R | ATTCCCAAAAGAGCGATAAACATTC |
| gadC-RT F | GCAGGGGTAATCCAAACTTTTCAAA |
| gadC-RT R | ATCCCTCTAGAAGGACCAACAACCC |
| ABC-RT F | AGTCACTGGCGATATTAAATGGGGA |
| ABC-RT R | ACCTTTGAAAAGCATACGTCCAAGG |
| nah-RT F | TAGTGTGGGAATGTCAGCCGTGATA |
| nah-RT R | CTGTCAAATTGAACGGATATGGCAA |
| nha-RT F | GTATCGCAACGATTCTTTTGGTTCC |
| nha-RT R | GAATGAACATTCCGACCAAAAAGCT |
| busAB-RT F | CTGATGGAATGGTTTCTGCTTGGTT |
| busAB-RT R | ACCAGGCTCAATTCCTGTGATTGTT |
| busAA-RT F | GTTAATGGATGAAGCCTTTTCTGCG |
| busAA-RT R | TCGCAATACGATCACCAATACGAAG |
| gltP-RT F | GATTCCTGGGATTATCGTGATTTCG |
| gltP-RT R | TCCTACGGCTGGTAAAATATTTCGC |
| gltS-RT F | ACATTGGGATTAGTTGGTTTGGCTG |
| gltS-RT R | AATTCAAAGGGTGGATAATCTGGGG |
| gltQ-RT F | AGCAACAAAGGGTAGCTATTGCTCG |
| gltQ-RT R | TTAGCAAAGCCCATTTCATGAGTGA |
| arcD1-RT F | TTTCGTTGCGAATACAAATGCTGAT |
| arcD1-RT R | GATTCCGACGAAGACCCAAATCATA |
| arcD2-RT F | TGTCAAAGCCCCTGGTCTTGTTTAC |
| arcD2-RT R | CCAACTTGGCAATAGCTTTTGCTCT |
| tuf-RT F  tuf-RT R  gyrA-RT F  gyrA-RT R | GACTTCCCAGGTGACGATATTCCTG  ACGTCTTCGACTGGAAGAAGGAGTG  TTTGGTTTCAACATGTTGGCTATCG  CCTTCTAAGATATGTGCCCGTGCTT |
