# Supplementary material for: Expression of PprI from Deinococcus radiodurans Improves Lactic Acid Production and Stress Tolerance in Lactococcus lactis
Source: PLoS One. 2015 Nov 12;10(11):e0142918. doi: 10.1371/journal.pone.0142918 (PMC4643010; doi:10.1371/journal.pone.0142918)
Supplement: S2 Table — (DOC) [file pone.0142918.s002.doc]

| Locus | Annotation | Fold |
| --- | --- | --- |
|  |  | MG(V) MG(P) |
| gltS | arginine/ornithine ABC transporter substrate-binding protein | 1.701±0.121 5.245±0.096 |
| gltQ | glutamate ABC transporter ATP-binding protein; | 1.806±0.051 5.609±0.02 |
| gltP | glutamate ABC transporter permease | 1.874±0.11 3.717±0.011 |
| arcD_2_ | arginine/ornithine antiporter | 1.866±0.04 4.566±0.025 |
| arcD_1_ | arginine/ornithine antiporter | 1.831±0.06 4.883±0.075 |
| atpC | ATP synthase F0F1 subunit epsilon | 2.023±0.06 5.928±0.065 |
| busAA | glycine betaine/proline ABC transporter | 1.304±0.15 2.746±0.15 |
| busAB | glycine betaine-binding periplasmic protein; | 1.183±0.319 3.061±0.319 |
| ctrA | amino-acid transporter | 1.697±0.127 4.182±0.115 |
| gadC | glutamate/gamma-aminobutyrate antiporter | 2.297±0.07 4.096±0.105 |
| ctpE | cation transporter E1-E2 family ATPase | 1.958±0.04 5.173±0.081 |
| pacL | cation transporter E1-E2 family ATPase | 2.188±0.026 4.340±0.14 |
| nha | Na^+^/H^+^ antiporter; | 2.675±0.121 6.473±0.41 |
| nah | Na^+^/H^+^ antiporter | 3.506±0.41 8.56 ±0.121 |
| ABC | ABC transporter ATP-binding protein | 2.219±0.55 1.181±0.253 |
| tuf | Houskeeping gene | 1 1 |
| gyre | Houskeeping gene | 1 1 |

**S2 Table. Transcriptions of selected stress-related genes in MG(PprI^+^) under salt stress**
